# CB2 Receptor Activation Inhibits Melanoma Cell Transmigration through the Blood-Brain Barrier

**DOI:** 10.3390/ijms15058063

**Published:** 2014-05-08

**Authors:** János Haskó, Csilla Fazakas, Judit Molnár, Ádám Nyúl-Tóth, Hildegard Herman, Anca Hermenean, Imola Wilhelm, Yuri Persidsky, István A. Krizbai

**Affiliations:** 1 Institute of Biophysics, Biological Research Centre of the Hungarian Academy of Sciences, P.O. Box 521, Szeged H-6701, Hungary; E-Mails: hasko.janos@brc.mta.hu (J.H.); fazakas.csilla@brc.mta.hu (C.F.); molnar.judit@brc.mta.hu (J.M.); nyul-toth.adam@brc.mta.hu (A.N.-T.); wilhelm.imola@brc.mta.hu (I.W.); 2 Institute of Life Sciences, Vasile Goldis Western University of Arad, Arad 310414, Romania; E-Mails: maszatkaa@yahoo.com (H.H.); anca.hermenean@gmail.com (A.H.); 3 Department of Pathology and Laboratory Medicine, Temple University School of Medicine, Philadelphia, PA 19140, USA; E-Mail: yuri.persidsky@tuhs.temple.edu

**Keywords:** blood-brain barrier (BBB), cerebral metastasis, melanoma, cannabinoid, CB2

## Abstract

During parenchymal brain metastasis formation tumor cells need to migrate through cerebral endothelial cells, which form the morphological basis of the blood-brain barrier (BBB). The mechanisms of extravasation of tumor cells are highly uncharacterized, but in some aspects recapitulate the diapedesis of leukocytes. Extravasation of leukocytes through the BBB is decreased by the activation of type 2 cannabinoid receptors (CB2); therefore, in the present study we sought to investigate the role of CB2 receptors in the interaction of melanoma cells with the brain endothelium. First, we identified the presence of CB1, CB2(A), GPR18 (transcriptional variant 1) and GPR55 receptors in brain endothelial cells, while melanoma cells expressed CB1, CB2(A), GPR18 (transcriptional variants 1 and 2), GPR55 and GPR119. We observed that activation of CB2 receptors with JWH-133 reduced the adhesion of melanoma cells to the layer of brain endothelial cells. JWH-133 decreased the transendothelial migration rate of melanoma cells as well. Our results suggest that changes induced in endothelial cells are critical in the mediation of the effect of CB2 agonists. Our data identify CB2 as a potential target in reducing the number of brain metastastes originating from melanoma.

## 1. Introduction

The blood-brain barrier (BBB) forms a protective shield between the central nervous system (CNS) and the systemic circulation, thus contributing substantially to the maintenance of the homeostasis of the CNS. The BBB serves as a barrier not only to solutes but restricts the free movement of cellular elements between the two compartments as well [1,2]. The barrier properties are mainly provided by cerebral endothelial cells interconnected by a continuous line of tight junctions [3,4]. The BBB plays a critical role in the pathogenesis and outcome of a large number of neurological disorders including neurodegenerative diseases, inflammatory processes of the brain, traumatic injury, stroke or brain tumors. BBB has a special importance in the formation of brain metastases of malignant tumors. Since the CNS lacks a lymphatic system, the only way of metastatic cells to reach the brain is to cross one of the principal barriers protecting the brain: the blood-CSF barrier or the blood-brain barrier. Since brain metastases are life threatening pathologies and our therapeutic possibilities in case of already formed brain metastasis are very limited, an optimal strategy would be the inhibition of transmigration of metastatic cells through the BBB [5].

The cannabinoid system is known mainly for its psychoactive effects; however, it has been previously shown that its activation may induce anti-inflammatory and neuroprotective processes as well [6,7]. Cannabinoids exert their effects mainly through two receptor types: CB1 and CB2 which mediate distinct effects. The psychoactive effect is mediated by the type 1 cannabinoid receptors (CB1), whereas the anti-inflammatory effects are mainly mediated by the type 2 cannabinoid receptors (CB2) actions [8]. CB1 is expressed mainly in the CNS, whereas CB2 is expressed predominantly in cells of the immune system and hematopoietic cells. In the brain CB2 receptors are found primarily on microglia [9], but endothelial cells also express this type of receptor [10,11].

Induction of cannabinoid-like effects by substances which do not activate CB1 or CB2 suggested the existence of other cannabinoid receptors as well. One such receptor is GPR18 which has been shown to bind *N*-arachidonoyl glycine (NAGly), an endogenous metabolite of anandamide [12]. GPR55 and GPR119 have also been shown to mediate the effect of cannabinoids [13].

Recently, we have shown that activation of CB2 improves barriers properties of the endothelial layer by increasing the amount of tight junction proteins in the membrane fractions [14]. Moreover, CB2 activation reduces the amount of ICAM-1 and VCAM-1 expression induced by inflammatory mediators in cerebral vascular endothelial cells and attenuates the adhesion and transmigration of leukocytes through the BBB.

However, no information is available about the role of the cannabinoid system in the transmigration of metastatic cells through the BBB. Since normal melanocytes and malignant melanoma share signaling similarity with the brain [15,16], we expected melanoma cells to respond to cannabinoid signals. Therefore, in this study we investigated the expression of different cannabinoid receptors in cerebral endothelial cells and determined the role of CB2 activation in the adhesion of melanoma cells to the cerebral endothelium and transmigration of these cells through the BBB.

## 2. Results and Discussion

### 2.1. Expression of Cannabinoid Receptors and Cannabinoid-Like Receptors in Brain Endothelial Cells and Melanoma Cells

Besides their well-known psychotropic effects, cannabinoids are able to regulate a wide range of physiological and pathological processes, including inflammation [17], angiogenesis [18] or cancer [19,20]. Due to the psychotropic effect of CB1 activation research has been focused mainly on CB2 receptors. Previous studies have demonstrated the expression of CB2 receptors mainly in peripheral tissues, particularly in immune cells [21]. In the CNS expression of CB2 receptors has been detected in microglial and perivascular cells (for review see: [22]) in response to neuroinflammatory processes. Endothelial cells including human umbilical vein endothelial cells, pulmonary artery endothelial cells [23] and brain microvascular endothelial cells [24] have also been described to express CB2 receptors. However, the expression profile of other receptors which could mediate the effect of cannabinoids is less well known. Therefore, in the first step of our investigations we determined the expression pattern of cannabinoid and cannabinoid-like receptors in cerebral endothelial cells by using RT-PCR. Cannabinoids may influence endothelial cell-immune cell interactions; however, it is not known whether they can regulate endothelial cell-cancer cell interactions as well. Since melanoma has the highest propensity to form brain metastases, we have also investigated the cannabinoid receptor expression profile in melanoma cells.

**Figure 1.**
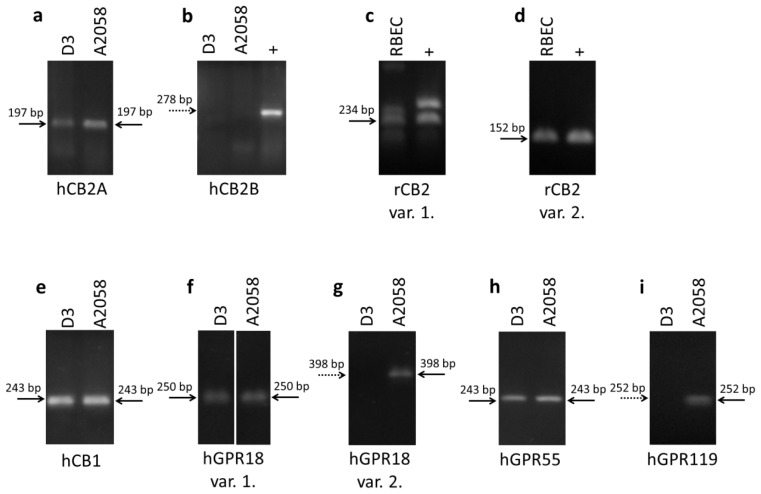
Expression of cannabinoid and cannabinoid-like receptors in brain endothelial and melanoma cells. RT-PCR was performed to determine the expression of CB2A and CB2B (positive control: HL-60) transcriptional variants of human CB2 receptor in hCMEC/D3 brain endothelial and A2058 melanoma cells (**a**,**b**); the expression of transcriptional variant 1 and 2 of rat CB2 receptor (**c**,**d**) in rat brain endothelial cells (RBECs) (positive control: rat spleen), the expression of CB1 receptor (**e**); transcriptional variant 1 and 2 of GPR18 (**f**,**g**); GPR119 (**h**) and GPR55 (**i**) in hCMEC/D3 human brain endothelial cells and A2058 melanoma cells. Dotted arrows indicate the absence of specific bands.

Our results demonstrate that hCMEC/D3 human brain endothelial cells and A2058 human melanoma cells express the CB2A transcriptional variant of the CB2 receptor but not the CB2B (Figure 1a,b). CB2A and CB2B differ in their untranslated 5′ region indicating that the two variants may differ in regulatory aspects only. CB2A has higher expression in the testis and the brain, whereas CB2B is expressed in higher amounts in peripheral tissues [25]. Primary rat brain endothelial cells were found to express variants 1 and 2 of CB2 receptor (Figure 1c,d). In addition hCMEC/D3 cells express CB1 receptor, GPR18 (transcriptional variant 1, but not transcriptional variant 2) and GPR55, whereas the presence of GPR119 was not detectable in this cell line (Figure 1e–i). Furthermore, we detected the presence of CB1, GPR18 transcriptional variants 1 and 2, GPR55 and GPR119 in A2058 melanoma cells (Figure 1e–i). These results complement previous data demonstrating the expression of CB1 and CB2 in melanoma cells [26].

Initially, GPR18 has been detected in testis and spleen [27], but it is expressed in the thymus, peripheral white blood cells and small intestine as well. No expression has been detected so far in the brain [28]. Under culture conditions it is expressed in metastatic melanoma [29], BV2 murine microglial cells [12] and HEC-1B human endometrial cells [30] as well. To our knowledge this is the first report of its expression in cerebral endothelial cells. However, further experiments are needed to understand the role of GPR18 splice variants.

GPR55 is another cannabinoid-like receptor which can be activated by *N*-arachidonoyl-serine, an endocannabinoid-like lipid with structural similarities to the endocannabinoid *N*-arachidonoyl ethanolamide (anandamide) [31]. *N*-arachidonoyl-serine promotes proliferation, migration and tube formation of primary human dermal microvascular endothelial cells, an effect at least partially mediated by GPR55 [32]. In the brain this receptor regulates microglia migration [33] and can be involved in microglia-mediated neuroprotection [34]. The role of GPR55 in cerebral endothelial cells is still unknown. In addition, GPR55 is expressed in malignant tumors as well including cholangiocarcinoma cells, melanoma cells or human squamous cell carcinomas. The role of GPR55 activation seems to be cell type specific: while in cholangiocarcinoma cells and melanoma cells activation of GPR55 has anti-proliferative or even toxic effects [35,36] in human squamous cell carcinomas drives skin carcinogenesis [37].

Furthermore, we have detected the expression of GPR119 in A2058 cells which is in accordance with previous results demonstrating the presence of this receptor in melanoma cells [29]. GPR119 was found in pancreatic and intestinal tissues and in some brain regions (for review see: [38]), but cerebral endothelial cells seem not to express this receptor.

### 2.2. Effect of CB2 Activation on the Adhesion of Melanoma Cells to the Brain Endothelium

The cannabinoid system plays an important role in different aspects of cancer formation [39]. However, its role in brain metastasis formation is less well understood. Steps of brain metastasis formation include adhesion of cancer cells to the cerebral endothelium, transmigration through the BBB and proliferation of tumor cells in the brain parenchyma. The first two steps are characterized by complex interactions of endothelial cells with metastatic cells [40–42].

**Figure 2.**
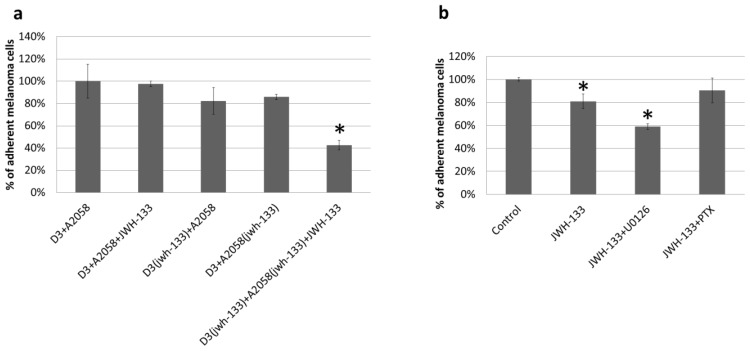
Effect of CB2 activation on the attachment of melanoma cells on the brain endothelium. Results are represented as % control (*i.e.*, D3 + A2058) and given as mean ± SD. *N* = 3. * *p* < 0.05 as assessed by ANOVA and Bonferroni’s *post-hoc* test. (**a**) D3(jwh-133) and A2058(jwh-133) represent cells pre-treated with 10 μM JWH-133 for 4 h. D3 + A2058 + JWH-133 denotes cells treated with 10 μM JWH-133 during the 90 min adhesion assay; (**b**) JWH-133 (10 μM), U0126 (10 μM) and PTX (100 ng/mL) were applied in pre-treatment of both cell types and treatment during the adhesion assay.

We tested whether activation of the CB2 receptor with its agonist JWH-133 can affect the attachment of melanoma cells to brain endothelial cells. Treatment of human brain endothelial cells (hCMEC/D3) or human melanoma cells (A2058) with JWH-133 during the adhesion assay did not alter adhesion of melanoma cells to the endothelium (Figure 2a). However, a 4 h long pre-treatment of both endothelial cells and melanoma cells with JWH-133 and treatment with the same compound during the adhesion assay significantly reduced the number of adhered melanoma cells compared to the untreated control (Figure 2a). This is not surprising, since JWH-133 was shown to exert its TEER elevating effect starting from 4 h [14]. In order to determine the cell-type specific role of CB2 activation we pre-treated either melanoma or endothelial cells with JWH-133 and performed the adhesion experiment in the absence of the CB2 agonist. Activation of CB2 receptors of cerebral endothelial cells or melanoma cells with JWH-133 slightly reduced the adhesion of melanoma cells to the confluent layer of brain endothelial cells. These results suggest that activation of CB2 receptors on both endothelial cells and melanoma cells contribute to the adhesion reducing effect of JWH-133 (Figure 2a).

CB2 receptors exert their effect through Gi/Goα subunits and are also coupled to the MAPK-ERK pathway [43]. In order to explore which pathway is responsible for the observed impact of CB2 activation on the melanoma cell adhesion, adhesion experiments were performed in the presence of PTX as a Gi/Goα inhibitor and U0126 as a MEK inhibitor. PTX blocked the effect of the CB2 agonist whereas U0126 did not reverse the adhesion reducing effect of CB2 activation (Figure 2b). This indicates that CB2 exerts its anti-adhesive effect mainly through activation of Gi/Goα.

Previously we have shown that activation of CB2 receptors reduces endothelial-immune cell interactions, especially under inflammatory conditions. Similarly to leukocytes and monocytes, we also found a reduction in the adhesion of A2058 melanoma cells to the cerebral endothelium. However, the reduction could be observed only when both endothelial cells and melanoma cells were pre-treated with the CB2 agonist. CB2 signaling is mainly mediated by Gi/Goα subunits, but the MAPK-ERK pathway can also be activated by CB2. Both signaling pathways are active in cerebral endothelial cells [44,45]. The Gi inhibitor PTX completely abolished the effect of CB2 stimulation whereas inhibition of the MAPK-ERK pathway had an additive effect to JWH-133, indicating that the adhesion reducing effect of CB2 activation is rather Gi than MAPK-ERK signaling dependent.

### 2.3. Effect of CB2 Activation on the Transmigration of Melanoma Cells through Brain Endothelial Cell Layers

**Figure 3.**
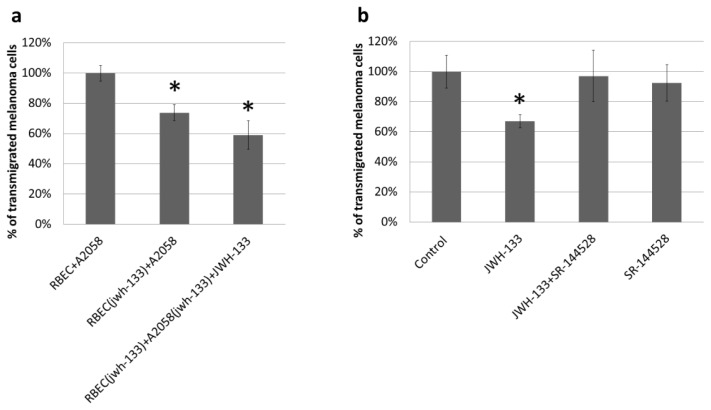
Effect of CB2 activation on the transendothelial migration of melanoma cells. Results are represented as % control (*i.e.*, RBEC + A2058) and given as mean ± SD. *N* = 3. * *p* < 0.01 (compared to control) as assessed by ANOVA and Bonferroni’s *post-hoc* test. (**a**) RBEC(jwh-133) represents endothelial cells pre-treated with 10 μM JWH-133 for 4 h. RBEC(jwh-133) + A2058(jwh-133) + JWH-133 denotes that both endothelial and melanoma cells were pre-treated with 10 μM JWH-133 for 4 h and treated with 10 μM JWH-133 during the 5 h transmigration assay; (**b**) JWH-133 (10 μM) and SR-144528 (10 μM) were applied in pre-treatment of both cell types and treatment during the transmigration assay.

Our next set of experiments was designed to understand whether CB2 activation can interfere with the transendothelial migration of melanoma cells as well. Transendothelial migration of A2058 cells was tested on primary brain endothelial cells (RBECs) cultured on filter inserts with 8 μm pore size to allow migrating cells to reach the bottom of the filter. Pre-treatment of brain endothelial cells with JWH-133 reduced the migration rate of melanoma cells (Figure 3a), suggesting that changes induced in endothelial cells by CB2 agonists are critical in the mediation of the effect of CB2 agonists. One such change could be the improvement of barrier properties in response of CB2 activation, since CB2 agonists increase the TEER of brain endothelial cells [14]. This may have its molecular background in the increase of claudin-5 expression in cerebral endothelial cells in response to CB2 activation. Furthermore, cannabinoids have been shown to downregulate adhesion molecules like ICAM or VCAM [46] and matrix metalloproteinases [47] which could also contribute to a reduced transmigration.

A more potent reduction in the number of transmigrated melanoma cells was observed when both cell types were pre-treated with the CB2 agonist, which was also applied during the transmigration (Figure 3a). The CB2 reverse agonist SR-144528 completely blocked the effect of JWH-133 on the transendothelial migration of A2058 melanoma cells, proving the CB2 specific effect of JWH-133 (Figure 3b). SR-144528 alone did not have any effect on the transmigration.

## 3. Experimental Section

### 3.1. Reagents

The selective CB2 agonist JWH-133 solution (diluted in Tocrisolve) was purchased from Tocris. The selective CB2 inverse agonist SR-144528 (dissolved in ethanol) [48,49] was from Santa Cruz, the MEK1/2 inhibitor U0126 was from Cell Signaling and the Gi/Goα inhibitor pertussis toxin (PTX) was from Sigma-Aldrich (Budapest, Hungary).

### 3.2. Cell Culture

The human microvascular cerebral endothelial cell line (hCMEC/D3; D3 for brevity) was maintained in EBM-2 medium (Lonza, Basel, Switzerland) supplemented with EGM-2 growth factors (Lonza) and 5% FBS. The A2058 human amelanotic melanoma cell line (obtained from the European Collection of Cell Cultures) was cultured in MEM (Sigma) and 5% FBS (Sigma). Primary rat brain endothelial cells (RBECs) were isolated from 2 week old rats, as described previously [50]. Briefly, after removal of meninges cerebral cortices were cut into small pieces and digested in two steps with collagenase and collagenase/dispase followed by centrifugation on percoll gradient. Isolated microvessels were plated on fibronectin/collagen-coated dishes. Endothelial cells growing out of the microvessels were cultured in DMEM/F12 (Life Technologies, Budapest, Hungary), 10% plasma-derived serum (PDS, First Link) and growth factors. In the first two days, 4 μg/mL puromycin was added to remove contaminating cells.

### 3.3. RT-PCR

Total RNA was isolated using TRIzol reagent (Life Technologies) following the manufacturer’s recommendations. RNA was transcribed into cDNA using the SuperScript III reverse transcription kit (Life Technologies). The amplification was performed on a BioRad iQ5 instrument using Maxima SYBR Green Mix (Fermentas, Vlinius, Lithuania) under the following conditions: 40 cycles of 95 °C for 15 s, 56 °C for 30 s, 72 °C for 30 s. Primer pairs used for amplification are summarized in Table 1. PCR products were electrophoresed on 1.5% agarose gels stained with ethidium bromide.

### 3.4. Adhesion Assay

D3 human brain endothelial cells were grown until confluency in 12-well plates. Both endothelial cells and melanoma cells were pre-treated with the mentioned drugs for 4 h. After pre-treatment, A2058 melanoma cells were fluorescently labeled using Oregon Green^®^ 488 carboxylic acid diacetate succinimidyl ester (OG, Life Technologies) using the protocol supplied by the manufacturer. 10^5^ melanoma cells/well were loaded onto the endothelial monolayer in serum-free medium and left for 90 min. After washing, cells were fixed using ethanol/acetic acid (95/5) at −20 °C for 5 min. Melanoma cells attached to endothelial cells were photographed and counted using the Image-Pro Plus software (Media Cybernetics, Rockville, MD, USA).

### 3.5. Transmigration Assay

For transmigration experiments primary brain endothelial cells were used because of their superior barrier characteristics. RBECs were passed onto fibronectin/collagen-coated 8 μm pore size filter inserts (Millipore, Budapest, Hungary). After reaching confluence, endothelial cells were supplemented with 550 nM hydrocortisone, 250 μM CPT-cAMP (Sigma) and 17.5 μM Ro 20-1724 (Sigma) from the apical side and astrocyte-conditioned medium from the basolateral side for 24 h. 10^5^ OG-labeled melanoma cells were plated into the upper chamber, onto the endothelial monolayer in serum-free medium and left for 5 h. The lower compartment was loaded with serum-free medium containing 100 μg/mL type I collagen. Cells were fixed with ethanol/acetic acid. Cells from the upper compartment were wiped off with a cotton swab. Transmigrated melanoma cells were counted using the Image-Pro Plus software.

## 4. Conclusions

Taken together, we have clarified the expression of classical and potential cannabinoid receptors on cerebral endothelial and melanoma cells and shown that activation of CB2 receptors reduces adhesion and transmigration of melanoma cells through the cerebral endothelium. This identifies CB2 as a potential target in reducing the number of brain metastases originating from melanoma.

## Figures and Tables

**Table 1.** Primers used for RT-PCR.

Name	Forward primer	Reverse primer	Product size
hCB2A	TCGCGCGTTGTAAGTGCACAG	TCGGCTGGAGCTCGGTGAGT	197
hCB2B	TGCCCAGCCACCCACAACACA	TATGAGGGCTTCCGGCGGAGT	278
rCB2 var. 1.	AGGCCAGACCTCCTCTCACCC	CCCGCCATGGACAGACAGGC	234
rCB2 var. 2.	CGAGGCCACCCAGCAAACAT	GGGTTGAACTCCAAGCCGCCA	152
hCB1	GTTCCTCACAGCCATCGACA	AGAAGCAGTACGCTGGTGAC	243
hGPR18 var. 1.	AAAGTCAGCCCAGCACCAACTCC	CAGCTGCTCTACTTCAGTGGTTCAC	250
hGPR18 var. 2.	TCCGACGCCAAGCGTTACACTG	TACCGTGGTTCTCTTCTTGGTGGT	398
hGPR55	CTGCAGGACACCACGATCTC	GATCCCTGAACACTGGGTGG	243
hGPR119	CGCAGCTGCCTCTGTCCTCA	ACGCAGGAGAGGGTCAGCAC	252
